# Effect of Dietary Supplementation with Red-Fleshed Dragon Fruit (*Hylocereus polyrhizus*) Juice By-Product Extract (HJBE) on Growth Performance, Digestive Enzyme Activity, Antioxidant Status, Immune Parameters, and Disease Resistance Against *Edwardsiella tarda* in Juvenile Red Seabream (*Pagrus major*)

**DOI:** 10.3390/antiox14121472

**Published:** 2025-12-08

**Authors:** Da Ye Kang, Tae Hoon Lee, Hwa Yong Oh, Young Wook Kim, Do Hyun Kwon, Hee Sung Kim, Seong-Mok Jeong

**Affiliations:** 1 Department of Marine Biology and Aquaculture, Gyeongsang National University, Tongyeong 53064, Republic of Korea; ekdp3292@gnu.ac.kr (D.Y.K.); xogns2357@gnu.ac.kr (T.H.L.); oho1203@gnu.ac.kr (H.Y.O.); kimmangkkong@gnu.ac.kr (Y.W.K.); wngsod@gnu.ac.kr (D.H.K.); 2 Aquafeed Research Center, National Institute of Fisheries Science, Pohang 37517, Republic of Korea

**Keywords:** *Hylocereus polyrhizus*, *Pagrus major*, juice by-product extract, antioxidant defense, immune response

## Abstract

This study investigated the effects of dietary supplementation with *Hylocereus polyrhizus* juice by-product extract (HJBE) on growth performance, digestive enzyme activity, antioxidant status, immune response, and disease resistance against *Edwardsiella tarda* in juvenile *Pagrus major*. The HJBE, prepared from juice-processing residues, contained measurable levels of bioactive compounds, including vitamin C, total phenolics, and flavonoids, and exhibited noticeable antioxidant activity. Five experimental diets containing 0 (control), 0.5, 1.0, 2.0, and 3.0 g/kg HJBE were fed to juvenile red seabream (initial weight of 7.0 ± 0.01 g) for eight weeks. Fish fed the diet containing 1.0 g/kg HJBE (HJBE1) showed significantly higher final weight, weight gain, specific growth rate, feed efficiency, and protein efficiency ratio compared with other groups. Trypsin activity in intestine was also significantly enhanced in the HJBE1 group, suggesting improved feed utilization. Whole-body composition and hematological indices did not differ among treatments. Plasma antioxidant parameters showed a dose-dependent trend, with catalase and glutathione levels lower at inclusion levels of 0.5–1.0 g/kg. Serum lysozyme activity and immunoglobulin M concentrations were significantly higher in fish fed HJBE1 compared to the controls, corresponding to improved survival after *E. tarda* challenge. These findings suggest that even moderate levels of bioactive compounds in HJBE can beneficially influence antioxidant homeostasis, immune defense, and growth performance. In conclusion, dietary inclusion of 1.0 g/kg HJBE effectively improved growth, digestive physiology, antioxidant balance, and disease resistance in juvenile *P. major*.

## 1. Introduction

Global aquaculture has expanded significantly over the past two decades, becoming a key contributor to food security, economic growth, and livelihoods worldwide [1]. However, this rapid expansion has also increased the exposure of farmed fish to various environmental and biological stressors. These include pathogen outbreaks, oxidative stress, and imbalances in nutritional inputs, all of which can compromise fish health and performance [2]. Marine finfish in the juvenile stage are especially vulnerable, as their immune and antioxidant defense mechanisms are not yet fully developed, making them more susceptible to infections and stress-induced damage [3,4]. Among these species, red seabream (*Pagrus major*) is one of the most commercially important fish in East Asian aquaculture. It is widely cultured in countries such as Japan, China, and South Korea due to its high market demand, favorable growth characteristics, and consumer acceptance. In South Korea alone, the annual aquaculture production of *P. major* has consistently exceeded 6474 metric tons in recent years, highlighting its significance within the national seafood industry [5]. Ensuring the health and survival of juvenile *P. major* is therefore essential for stable and profitable aquaculture operations. This necessity has prompted increasing interest in dietary approaches that can enhance fish immunity, growth, and resistance to disease without the use of antibiotics or synthetic growth promoters.

Among the natural alternatives, plant-derived bioactive such as polyphenols, flavonoids, and dietary antioxidants have shown promising effects in modulating fish immune responses, enhancing digestive physiology, and improving oxidative stress resilience [6,7]. Growing attention has been paid to the reutilization of agro-industrial by-products such as fruit peels, pulp residues, and seed husks, which are often discarded during food processing. These by-products, once considered waste, are now being explored as cost-effective and sustainable feed ingredients in aquaculture [8]. Recent studies have demonstrated that such by-products may retain substantial quantities of functional compounds with bioactive properties, including antioxidants, fiber, and phytochemicals, making them valuable alternatives to conventional feed components [9,10,11]. To improve the bioavailability and efficacy of these functional compounds, extract-based formulations have gained momentum. Extraction allows for the concentration and stabilization of specific bioactive such as polyphenols, flavonoids, and vitamins, thus enhancing their physiological activity when incorporated into aquafeeds [6,12,13]. Unlike raw plant materials, extracts provide a more predictable and potent source of phytochemicals, which can more effectively modulate growth, immune response, and antioxidative defense in fish.

A notable example of a promising yet underutilized agricultural by-product is derived from the Cactaceae family, particularly *Hylocereus polyrhizus*, commonly known as red-fleshed dragon fruit. This fruit is widely cultivated in tropical and subtropical regions for its vibrant red pulp, appealing flavor, and nutritional richness. It contains a wide range of bioactive compounds, including vitamin C, betalains, anthocyanins, and various phenolic constituents, which contribute to its high antioxidant potential [14,15]. During juice production, considerable amounts of residual material such as peel, seeds, and fibrous pulp are typically discarded, even though these by-products still contain a significant proportion of the fruit’s phytochemicals. Recent studies have demonstrated that extracts obtained from these juice by-products exhibit strong antioxidant and antimicrobial activity in vitro [16,17,18]. While most of these findings have been reported in the context of food science and human health, such results suggest that juice by-product extracts from *H. polyrhizus* (HJBE) may hold potential as sustainable and functional additives in aquafeed formulations. Exploring the application of HJBE in fish diets could therefore offer a dual benefit: enhancing fish health and performance, while contributing to the valorization of fruit-processing waste. Nevertheless, studies evaluating the effects of supplementing diets with HJBE for juvenile *P. major* remain limited.

Therefore, the present study aimed to evaluate the effects of dietary supplementation with different inclusion levels of HJBE on juvenile *P*. *major*. The investigation focused on multiple physiological parameters, including growth performance, feed utilization, digestive enzyme activities, antioxidant status, and non-specific immune responses. In addition, the study assessed the fish’s resistance to *Edwardsiella tarda*, a major bacterial pathogen in marine aquaculture.

## 2. Materials and Methods

### 2.1. Preparation of HJBE and Chemical and Antibacterial Activity Analysis

By-products of *H. polyrhizus* juice processing, including peels, seeds, and residual pulp, were obtained from fresh fruit purchased in a private juice store (Jeju-si, Jeju-do, Republic of Korea). The fruits were initially rinsed with water and juiced using a commercial extractor (H-300L-DBFC03, Hurom Co., Ltd., Seoul, Republic of Korea). The collected by-products were then dried at 20 °C for 48 h using an agricultural product dryer (KED-M07D1, Kiturami Co., Ltd., Seoul, Republic of Korea), ground using a kitchen blender, and stored at 4 °C until further use. For extraction, the powder was mixed with 80% ethanol at a 1:10 (*w*/*v*) ratio and shaken intermittently at room temperature for 48 h. The mixture was filtered through Whatman No. 1 filter paper, and the filtrate was concentrated under reduced pressure at 40 °C using a rotary evaporator. The resulting concentrate was freeze-dried and stored at −20 °C until further analysis and inclusion in the experimental diets.

Vitamin C concentration in HJBE was determined by high-performance liquid chromatography (HPLC) using an Agilent 1200 Series system equipped with a UV detector set at 254 nm (Agilent Technologies, Anaheim, CA, USA). The mobile phase consisted of 0.05 M KH_2_PO_4_ buffer at pH 2.8, delivered at a flow rate of 1.0 mL/min. Samples were homogenized in 10% cooling metaphosphoric acid, centrifuged at 3000× *g* for 20 min, and the resulting supernatants were filtered through 0.45 μm syringe filters (Sartorius, Göttingen, Germany) prior to HPLC injection.

Total phenolic content was measured using the Folin–Ciocalteu colorimetric method [19]. Briefly, 1 mL of Folin–Ciocalteu reagent was added to 50 μL of the sample and vortexed, followed by the addition of 1 mL of 10% sodium carbonate solution after 3 min. The mixture was incubated for 60 min at room temperature, and absorbance was measured at 700 nm using a spectrophotometer. Gallic acid was used as the calibration standard. Total flavonoid content was determined following the method of [20], based on the reaction with aluminum nitrate and potassium acetate, and absorbance was measured at 415 nm after 40 min in the dark. Quercetin was used as the reference standard.

Antioxidant activity of HJBE was evaluated using both ABTS and DPPH radical scavenging assays. ABTS radical cations were generated by incubating 7 mM ABTS with 2.4 mM potassium persulfate for 16 h in the dark. The solution was diluted to an absorbance of 1.5 at 414 nm before use. For the assay, 50 µL of HJBE or ascorbic acid (positive control) was mixed with 100 µL of the ABTS working solution and incubated for 5 min at room temperature, after which absorbance was recorded at 414 nm. For the DPPH assay, a 150 µM solution of DPPH in methanol was mixed with 80 µL of sample or ascorbic acid, and the mixture was incubated for 10 min at room temperature before measuring absorbance at 525 nm using a microplate reader (SpectraMax^®^ M2/M2e, Sunnyvale, CA, USA). IC_50_ values were calculated for both assays.

The results of the chemical analysis are summarized in Table 1. HJBE contained 83.5 mg/100 g of vitamin C, 367.5 mg GAE/100 g of total phenolics, and 6.9 mg QE/g of flavonoids. In the antioxidant assays, HJBE demonstrated dose-dependent radical scavenging activity, with IC_50_ values of 657.3 µg/mL and 713.5 µg/mL for DPPH and ABTS, respectively.

### 2.2. Experimental Diet Preparation

A total of five experimental diets were formulated to evaluate the effects of different inclusion levels of HJBE (Table 2). The diets were designated as HJBE0 (0 g/kg, control), HJBE0.5, HJBE1, HJBE2, and HJBE3, corresponding to 0.5, 1.0, 2.0, and 3.0 g/kg HJBE supplementation, respectively, based on dry matter. To prepare the diets, all dry feed ingredients were first weighed accurately and premixed to ensure even distribution. Lipid sources, including fish oil and soybean oil, were then added to the dry mixture. To achieve the proper consistency for pelleting, distilled water was gradually introduced. For HJBE-containing diets, the extract was blended with the water prior to mixing to ensure uniform dispersion throughout the feed. Once the dough reached the desired texture, it was pelletized using a feed chopper fitted with a 3.0 mm die (SL Machinery, Incheon, Republic of Korea). The freshly formed pellets were spread evenly and dried at a constant temperature of 20 °C for 48 h using an agricultural dryer (Model KED-M07D1, Kiturami Co., Ltd., Seoul, Republic of Korea). Dried pellets were sealed in polyethylene bags and stored at −20 °C until the feeding trial commenced.

### 2.3. Feeding Trial Condition and Design Experiment

Juvenile *P. major* were obtained from a commercial hatchery (Tongyeong, Gyeongsangnam-do, Republic of Korea). Upon arrival, the fish were transferred to the Marine Bio-Education and Research Center at Gyeongsang National University (Tongyeong, Gyeongsangnam-do, Republic of Korea) and held in a flow-through seawater system for acclimation. During a two-week acclimation period, the fish were fed a commercial extruded pellet diet (Jeil Feed Co., Haman, Republic of Korea; containing 52% crude protein and 10% crude lipid) twice daily. Following a 24 h fasting period, a total of 525 juveniles with an initial average weight of 7.0 ± 0.01 g were randomly distributed into 15 rectangular tanks (200 L capacity), with 35 fish per tank. Each of the five dietary treatments was assigned to three replicate tanks. Fish were reared in a continuous flow-through seawater system equipped with individual aeration in each tank. Water temperature (21.4 ± 0.45 °C), salinity (32.2 ± 0.26 psu), and dissolved oxygen (7.5 ± 0.81 mg/L) were monitored daily using a multiparameter water quality meter (YSI Pro Plus, YSI Inc., Yellow Springs, OH, USA). A 9 h light and 15 h dark photoperiod (09:00–18:00 light phase) was maintained using overhead LED fluorescent lamps. The experimental diets were administered by hand to apparent satiation twice daily, at 09:00 and 17:00, for a duration of 8 weeks. Feed intake was recorded daily for each tank. Feces were removed once daily at 13:00 using a siphon to maintain water quality. Fish health and behavior were observed daily, and any mortalities were promptly recorded and removed.

### 2.4. Growth Performance Parameters

At the end of the 8-week feeding trial, all experimental fish were fasted for 24 h. The total body weight of all fish in each tank was recorded at the beginning and end of the trial. Subsequently, 10 fish were anesthetized using tricaine methanesulfonate (MS-222; Sigma-Aldrich, St. Louis, MI, USA) at a concentration of 150 ppm prior to sampling and weighing. Growth performance and feed utilization parameters were calculated on a per-tank basis using the following standard formulas:

Survival (SR, %) = (number of fish at the end of the trial/number of fish at the beginning of the trial) × 100

Weight gain (WG, g/fish) = final body weight − initial body weight

Specific growth rate (SGR, %/day) = [ln final weight of fish − ln initial weight of fish]/days of feeding × 100

Feed consumption (FC, g/fish) = total dry feed intake/fish

Feed efficiency (FE) = WG of fish/feed consumed

Protein efficiency ratio (PER) = WG of fish/protein consumed

Condition factor (CF) = (body weight × 100)/(total length)^3^

Hepatosomatic index (HSI, %) = (Liver weight/whole − body weight) × 100

Viscerosomatic index (VSI, %) = (Viscera weight/whole − body weight) × 100

### 2.5. Sample Collection

For digestive enzyme analysis, three fish were randomly selected from each tank. These individuals were the same fish previously measured for growth performance and feed utilization to ensure consistency across physiological and biochemical assessments. The abdominal cavity was opened, and the entire intestine was carefully excised and rinsed with ice-cold phosphate-buffered saline (PBS, pH 7.4). The rinsed intestine was then immediately stored at −80 °C until digestive enzyme analysis. For blood biochemical, antioxidant, and immune parameter analyses, five fish were randomly selected from each tank, and blood was drawn from the caudal vein using heparinized or non-heparinized syringes depending on the intended analysis. Blood collected in heparin-coated syringes was centrifuged at 7000 rpm for 15 min at 4 °C to obtain plasma for biochemical and antioxidant assays. Blood collected with non-coated syringes was allowed to clot for 30 min at 4 °C and centrifuged at 3000× *g* for 5 min to obtain serum for lysozyme and immunoglobulin M (IgM) analyses. All plasma and serum samples were aliquoted and stored at −80 °C until analysis. For whole-body proximate composition, the same five fish previously measured for growth performance and feed utilization were sampled.

### 2.6. Digestive Enzyme Activity

Intestinal tissues were homogenized in 10 volumes (*w*/*v*) of ice-cold 0.86% physiological saline using a Tissue Lyser II homogenizer (QIAGEN, Velno, The Netherlands) operated in an ice bath to prevent enzymatic degradation. The homogenates were then centrifuged at 13,000 rpm for 10 min at 4 °C, and the resulting supernatants were collected for enzymatic analysis. The activities of amylase, trypsin, and lipase were quantified using commercial colorimetric assay kits (MyBioSource Company, San Diego, CA, USA), following the manufacturer’s protocols. Enzyme activities were normalized to protein content and expressed as milliunits per milligram of protein (mU/mg protein).

### 2.7. Hematological Index Analysis

The collected plasma was used to determine biochemical parameters. Aspartate aminotransferase (AST), alanine aminotransferase (ALT), total cholesterol (T-CHO), total protein (TP), and glucose (GLU) concentrations were measured using an automated clinical chemistry analyzer (Fuji Dri-Chem NX500i; Fujifilm, Tokyo, Japan), following the manufacturer’s guidelines.

### 2.8. Antioxidant Enzyme Parameter Analysis

The activities of superoxide dismutase (SOD) and catalase (CAT), as well as the concentration of glutathione (GSH) in plasma, were measured using commercial assay kits (Cayman’s Assay Kits, Cayman Chemical, Ann Arbor, MI, USA). All assays were performed according to the manufacturer’s protocols. Absorbance readings were taken using a spectrophotometer (MULTISKAN GO, Thermo Scientific, Vantaa, Finland), and results were expressed as U/mL (SOD), nmol/min/mL (CAT), and µM (GSH).

### 2.9. Immune Parameter Analysis

Lysozyme activity in serum was measured using a turbidimetric assay, as described by [21]. Briefly, 100 µL of serum was added to 1.9 mL of a *Micrococcus lysodeikticus* suspension (0.2 mg/mL; Sigma, St. Louis, MO, USA) prepared in 0.05 M sodium phosphate buffer (pH 6.2). The reaction mixture was incubated at 25 °C, and the absorbance at 530 nm was recorded over a 60 min period using a spectrophotometer (Thermo Fisher Scientific, Tewksbury, MA, USA). One unit of lysozyme activity was defined as the amount of enzyme causing a decrease in absorbance of 0.001 per minute. Serum IgM level was quantified using species-specific enzyme-linked immunosorbent assay (ELISA) kits: IgM was measured using a kit from Cusabio Biotech Co., Ltd. (Wuhan, China), following the manufacturers’ protocols. Results were expressed in mg/mL (IgM).

### 2.10. Proximate Body Composition

Whole-body samples were rinsed, chopped, and homogenized into a uniform paste for proximate composition analysis. The moisture, crude protein, crude lipid, and ash contents were analyzed following the standard procedures of the Association of Official Analytical Chemists [22]. Moisture content was determined by drying the samples in a drying oven at 105 °C for 24 h. Ash content was measured by incinerating the dried samples in a muffle furnace at 550 °C for 4 h. Crude protein was measured using the Kjeldahl method with a KD310-A-1015 KjelROC Analyzer (OPSIS Liquid LINE, Furulund, Sweden), and expressed as nitrogen content × 6.25. Crude lipid content was determined by the Soxhlet extraction method using a Soxtec™ extractor (ST 243; FOSS, Hillerod, Denmark).

### 2.11. Challenge Test Against E. tarda

At the end of the 8-week feeding trial, a bacterial challenge test was conducted to assess disease resistance against *E. tarda*. Ten fish were randomly selected from each tank and transferred to tanks (50 L rectangular tank) for the challenge. The *E. tarda* strain used in this study was obtained from the Korean Culture Collection of Aquatic Microorganisms, National Institute of Fisheries Science (Busan, Republic of Korea). Bacterial cultures were grown in tryptic soy broth (TSB) at 28 °C for 24 h and diluted to a final concentration of 1.0 × 10^6^ CFU/mL. Each fish was intraperitoneally injected with 0.1 mL of the bacterial suspension using a 1 mL syringe. During the 9-day challenge period, water temperature and dissolved oxygen were maintained at 23.7 ± 0.43 °C and 6.8 ± 0.41 mg/L, respectively. Fish were monitored at 6 h intervals, and mortality was recorded daily. Dead fish were promptly removed to prevent secondary infections. No feed was provided during the challenge phase.

### 2.12. Statistical Analysis

All percentage data, including survival rate and other ratio-based metrics, were arcsine square root transformed prior to statistical analysis. The results were presented as mean ± standard error (SE). The Kolmogorov–Smirnoff and Levene tests were used to determine the normality and homogeneity of variance of all variable data. Subsequently, data were analyzed using one-way analysis of variance (ANOVA) followed by Tukey’s honest significant difference (HSD) test to determine significant differences among treatment means. The significance level was set at *p* < 0.05. For the bacterial challenge test, Kaplan–Meier survival curves were generated and compared using both the Log-rank (Mantel–Cox) test and the Wilcoxon test to assess statistical differences in cumulative survival among groups during the 9-day post-infection period. All statistical analyses were conducted using SPSS software (version 27.0; IBM Corp., Armonk, NY, USA).

## 3. Results

### 3.1. Growth Performance and Feed Utilization

After 8 weeks of feeding, significant differences were observed among treatments in several growth performance and feed utilization parameters (Table 3). The final weight of fish fed the HJBE1 diet was significantly higher (*p* < 0.05) than those of other groups. Similarly, the highest WG and SGR were recorded in the HJBE1 group, both of which were significantly greater than those in the control (HJBE0) and higher-dose groups (*p* < 0.05). FI was significantly increased in fish fed HJBE0.5 and HJBE1 diets compared to the control (*p* < 0.05). FE and PER were also significantly improved in the HJBE1 compared to the HJBE3 group, which showed the lowest values (*p* < 0.05), respectively. Notably, the HJBE3 group showed a trend of decreased growth and nutrient utilization efficiency at the highest inclusion level. No significant differences were observed among dietary treatments for SR, CF, VSI and HSI (*p* > 0.05).

### 3.2. Digestive Enzyme Activities

The activities of digestive enzymes (amylase, trypsin, and lipase) in the intestine of juvenile *P. major* after the 8-week feeding trial are presented in Table 4. Among the measured enzymes, trypsin activity showed a significant difference among dietary treatments (*p* < 0.05). The fish fed the HJBE1 diet exhibited the highest trypsin activity, which was significantly greater than those of the control (HJBE0) and HJBE3 groups. Amylase and lipase activities showed no significant differences among treatments (*p* > 0.05).

### 3.3. Whole-Body Composition and Hematological Indices

No significant differences were observed in whole-body proximate composition among dietary treatments (*p* > 0.05) (Table 5). The moisture, crude protein, crude lipid, and ash contents remained consistent across all groups. Similarly, hematological indices including plasma TP, GLU, T-CHO, AST, and ALT showed no significant variations among the dietary groups (*p* > 0.05).

### 3.4. Antioxidant Enzyme Activities

The effects of dietary HJBE supplementation on plasma antioxidant enzyme activities in juvenile *P. major* are presented in Table 6. Among the measured antioxidant indicators, SOD activity showed no significant differences among dietary treatments (*p* > 0.05). In contrast, CAT activity differed significantly (*p* < 0.05), with the highest value observed in the HJBE0 group. CAT activity in HJBE0.5 and HJBE1 groups was significantly lower than that in the control, while the HJBE3 group exhibited CAT activity comparable to the control, indicating a partial restoration at higher HJBE levels (*p* < 0.05). Similarly, GSH concentration varied significantly across treatments (*p* < 0.05). Fish fed the HJBE0 and HJBE3 diets showed significantly higher GSH levels compared to those in the HJBE0.5 and HJBE1 groups, which recorded the lowest values (*p* < 0.05).

### 3.5. Immune Parameters

The effects of dietary HJBE supplementation on non-specific immune parameters of juvenile *P. major* are summarized in Table 7. Serum lysozyme activity and IgM levels were significantly influenced by dietary treatments (*p* < 0.05). Fish fed the HJBE1 diet exhibited the highest lysozyme activity and IgM concentrations, both of which were significantly higher than those in the control group (*p* < 0.05).

### 3.6. Challenge Test

**Figure 1:**
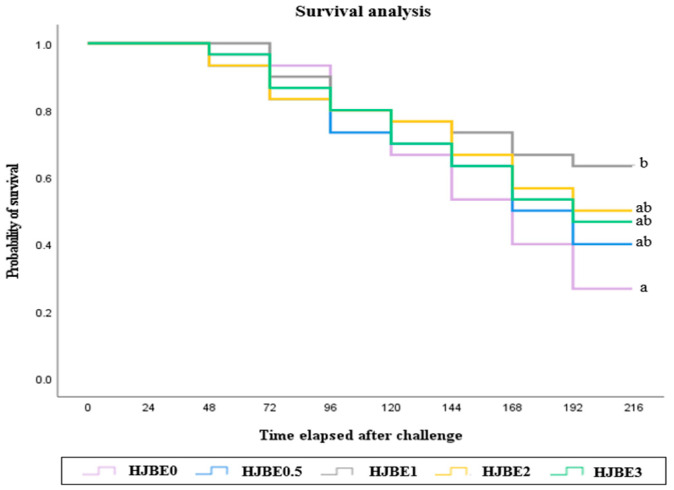
Survival of juvenile red seabream fed the experimental diets with different levels of *Hylocereus polyrhizus* juice by-product extract (HJBE) for 8 weeks, then artificially infected by *Vibrio harveyi* (means of triplicate ± SE). Different letters indicate significant differences (*p* < 0.001 for Log Rank and Wilcoxon tests).

The survival probabilities of juvenile *P. major* following *E. tarda* challenge are shown in Figure 1. Cumulative survival rates varied among dietary treatments over the 9-day post-injection period. Fish fed the HJBE1 diet exhibited the highest survival rate among all groups, followed by those fed HJBE0.5, HJBE2 and HJBE3, although the differences were not statistically significant between these three treatments (*p* > 0.05). In contrast, the HJBE0 (control) group showed the lowest survival probability, which was significantly different from HJBE1 (*p* < 0.05).

## 4. Discussion

The chemical analysis of the juice by-product extract from *H. polyrhizus* (HJBE) in the present study revealed that it contained considerable amounts of vitamin C (83.5 mg/100 g), total phenolics (367.5 mg GAE/100 g), and flavonoids (6.9 mg QE/g), along with strong DPPH and ABTS radical scavenging activities. These results confirm that the extract possesses high antioxidant potential, largely attributable to its abundant phenolic and flavonoid contents, which are well-known for their roles in neutralizing reactive oxygen species (ROS) and protecting biomolecules from oxidative damage [23]. In addition to these compounds, previous studies have reported that *H. polyrhizus* and its by-products contain other bioactive constituents, such as betalains (including betacyanins and betaxanthins), polysaccharides, and trace minerals [14,15,16,17]. Betalains, recognized for their potent antioxidant, anti-inflammatory, and antimicrobial activities, act as strong electron donors that scavenge free radicals and inhibit lipid peroxidation [18]. Similarly, flavonoids and phenolic acids have been shown to enhance digestive enzyme activity, support intestinal barrier function, and modulate immune responses through the activation of signaling pathways such as NF-κB and MAPK [10,13]. Vitamin C, another major component detected in this study, contributes to tissue repair, collagen synthesis, and redox regulation, playing a vital role in both antioxidant and immune defense systems [24]. The combination of these bioactive compounds’ vitamin C, phenolics, flavonoids, and betalains provides a mechanistic basis for the physiological improvements observed in the present study, including enhanced growth performance, balanced antioxidant status, and improved immune function in juvenile *P. major*. Therefore, the beneficial effects of HJBE can be interpreted as the synergistic outcome of its complex mixture of natural antioxidants and bioactive phytochemicals derived from *H. polyrhizus* juice by-products.

Dietary supplementation with the HJBE significantly influenced the growth performance and feed utilization efficiency of juvenile *P. major* in the present study. The fish fed the HJBE1 diet exhibited superior final weight, weight gain (WG), specific growth rate (SGR), feed efficiency (FE), and protein efficiency ratio (PER) compared with both the control and higher-HJBE-inclusion groups. These findings suggest that moderate inclusion of HJBE exerts a beneficial effect on growth and nutrient utilization, whereas excessive supplementation (e.g., HJBE3) may counteract these benefits. The growth-promoting effect observed at the 1.0 g/kg inclusion level could be attributed to the bioactive compounds present in HJBE, including vitamin C, phenolics, flavonoids, and betalains, which have been shown to enhance digestive physiology, antioxidant defense, and overall metabolic activity in fish [6,10,17]. Similar effects have been documented in other studies where plant-derived extracts rich in polyphenols improved feed efficiency and growth in marine species such as *Sparus aurata* and *Dicentrarchus labrax* by stimulating digestive enzyme secretion and improving intestinal health [12]. The increased trypsin activity observed in the HJBE1 group supports this interpretation, as enhanced proteolytic enzyme activity is closely associated with improved protein digestion and growth performance [2,11]. The superior FE and PER in the HJBE1 group also indicate that HJBE might have improved nutrient assimilation and reduced oxidative degradation of dietary components during metabolism. Bioactive constituents such as flavonoids and betalains have been reported to modulate intestinal morphology and nutrient transporter expression, leading to more efficient nutrient absorption [13,14]. Moreover, vitamin C, one of the dominant antioxidants in HJBE, may have contributed to enhanced collagen synthesis and tissue development, facilitating somatic growth [24]. Conversely, the decline in growth and feed utilization at higher inclusion levels (HJBE2 and HJBE3) could be due to an excessive intake of polyphenolic compounds or fiber components, which are known to exert antinutritional effects when consumed at high concentrations [6,7]. Polyphenols may form complexes with dietary proteins or digestive enzymes, reducing protein digestibility and amino acid absorption [8]. This biphasic response pattern a moderate stimulatory effect followed by a reduction at higher doses has been consistently reported for phytochemical-rich feed additives in aquaculture [25,26]. However, the absence of significant differences in hepatosomatic index (HSI) and viscerosomatic index (VSI) among treatments indicates that HJBE supplementation did not impose any physiological stress or hepatotoxic effects on the fish. This finding suggests that HJBE is a safe and functional feed additive at moderate inclusion levels. Similar non-toxic responses were reported in tilapia and seabass fed fruit peel extracts or other phytogenic by-products at low to moderate concentrations [8,11].

The activity of digestive enzymes is a key physiological indicator reflecting the capacity of fish to digest and utilize nutrients efficiently [27]. In the present study, dietary supplementation with HJBE significantly affected the intestinal trypsin activity of juvenile *P. major*, whereas amylase and lipase activities were not altered among treatments. The highest trypsin activity was observed in fish fed the HJBE1 diet (1.0 g/kg), which closely corresponded with the improved growth performance and feed utilization parameters in this group. This suggests that moderate supplementation of HJBE enhanced protein digestion and nutrient absorption by stimulating proteolytic enzyme activity. Trypsin plays a central role in the breakdown of dietary proteins into absorbable peptides and amino acids, directly influencing the PER and overall somatic growth [2,4]. The enhanced trypsin activity in the HJBE1 group may be attributed to the bioactive compounds in the extracts, particularly flavonoids and phenolics, which are known to stimulate digestive enzyme secretion and improve intestinal mucosal health [11,12]. Polyphenolic compounds can upregulate the expression of pancreatic digestive enzymes and modulate the activity of brush-border hydrolases, leading to improved feed conversion efficiency [8]. Furthermore, vitamin C and betalains contained in HJBE may contribute to maintaining enzyme stability and protecting intestinal cells from oxidative stress. Antioxidant protection in the gut environment is critical, as excessive ROS can impair enzyme activity and intestinal epithelial integrity [6,14]. By mitigating oxidative damage, HJBE may have indirectly preserved optimal enzyme functionality, ensuring efficient digestion and absorption of nutrients. The lack of significant changes in amylase and lipase activities indicates that HJBE specifically modulated proteolytic pathways rather than general digestive processes. This selective enhancement aligns with previous findings in seabream and tilapia fed plant-derived antioxidants, where protease activity increased while amylase and lipase remained stable [7,10]. Such enzyme-specific modulation could be linked to improved protein utilization efficiency rather than carbohydrate or lipid metabolism. At higher inclusion levels (≥2.0 g/kg), trypsin activity tended to decline, possibly due to the inhibitory effects of excessive polyphenols on enzyme systems. High concentrations of phenolic compounds have been reported to form complexes with digestive enzymes or dietary proteins, thereby reducing enzyme bioavailability and catalytic efficiency [6,28,29]. This biphasic trend activation at moderate levels and inhibition at excessive concentrations further supports the notion of an optimal dosage threshold for HJBE supplementation.

The whole-body proximate composition and hematological profiles of juvenile *P. major* were not significantly affected by dietary supplementation with HJBE. The consistency in body composition across treatments indicates that the extract did not alter the deposition of nutrients such as protein, lipid, or ash, despite the observed differences in growth and feed utilization. This suggests that the growth-promoting effect of HJBE, particularly at 1.0 g/kg inclusion, was due to enhanced FE and nutrient utilization rather than changes in nutrient allocation or storage patterns. Stable hematological parameters, including plasma total protein (TP), glucose (GLU), total cholesterol (T-CHO), aspartate aminotransferase (AST), and alanine aminotransferase (ALT), further support the notion that HJBE supplementation was physiologically safe and non-toxic to *P. major*. AST and ALT are commonly used indicators of hepatic integrity and metabolic stress [10], and their unchanged levels suggest that HJBE did not elicit hepatocellular damage or interfere with normal liver function. Likewise, the absence of variation in plasma GLU and TP reflects stable energy metabolism and homeostatic regulation, implying that the inclusion of HJBE did not impose metabolic burdens. Comparable findings have been reported in other fish species fed phytogenic additives or fruit by-product extracts. For instance, Refs [30,31] observed no alterations in whole-body composition or hematological indices in fish when moderate doses of plant extracts were included in the diet.

The antioxidant defense system in fish plays a critical role in maintaining cellular homeostasis and protecting tissues from oxidative damage caused by ROS [32]. In the present study, dietary inclusion of HJBE influenced certain plasma antioxidant parameters in juvenile *P. major*. While superoxide dismutase (SOD) activity did not show significant differences among treatments, catalase (CAT) activity and glutathione (GSH) concentrations varied significantly depending on the inclusion level of HJBE. Interestingly, CAT activity and GSH levels were relatively higher in the control (HJBE0) and high-dose (HJBE3) groups, whereas intermediate inclusion levels (HJBE0.5 and HJBE1) exhibited reduced values. This biphasic trend suggests that moderate supplementation with HJBE may have optimized the oxidative balance by reducing the need for excessive antioxidant enzyme activation. In other words, the decrease in CAT and GSH levels in the HJBE0.5 and HJBE1 groups could indicate a more stabilized redox state, where exogenous antioxidants provided by HJBE such as vitamin C, phenolics, flavonoids, and betalains effectively scavenged free radicals, thereby reducing the endogenous enzymatic antioxidant demand [6,14,17]. Similar findings have been reported in fish fed fruit-derived extracts, where exogenous antioxidant supplementation decreased endogenous enzyme activity due to lower oxidative stress levels [2,4]. Conversely, the elevated CAT and GSH activities observed in the control and high-dose groups may reflect compensatory physiological responses to oxidative stress. The control fish, lacking dietary antioxidants, likely experienced greater ROS accumulation, necessitating upregulation of endogenous antioxidant enzymes to maintain redox homeostasis. In contrast, the HJBE3 group may have encountered pro-oxidant pressure due to excessive intake of phenolic compounds, which at high concentrations can paradoxically promote oxidative reactions [7,12]. Such dual behavior of polyphenols acting as antioxidants at low levels but exhibiting pro-oxidant effects at high concentrations has been well documented in mammalian models and suggested in fish studies [32,33,34]. Therefore, the observed reduction in CAT and GSH at moderate inclusion levels likely reflects an optimal antioxidant equilibrium, wherein dietary antioxidants from HJBE effectively neutralized ROS and prevented oxidative activation of endogenous defense systems. This balanced antioxidant response could also have contributed to the improved growth and immune responses observed in the HJBE1 group, as oxidative stability supports better metabolic efficiency and immune cell function [35].

The immune responses and disease resistance of juvenile *P. major* were modulated by dietary supplementation with HJBE. Fish fed the HJBE1 diet exhibited the highest serum lysozyme activity and plasma immunoglobulin M (IgM) levels, which were consistent with their superior survival following *E. tarda* challenge. These findings indicate that moderate inclusion of HJBE (1.0 g/kg) effectively enhanced both innate and humoral immune defenses, conferring improved resistance to bacterial infection. Lysozyme and IgM are among the most crucial non-specific or specific immune effectors in fish, contributing to the first line of defense against pathogens [36,37]. Lysozyme directly hydrolyzes bacterial cell walls by cleaving β-(1,4)-glycosidic linkages in peptidoglycans, while IgM serves as the primary antibody in teleosts, facilitating pathogen recognition and opsonization [2,3]. The elevated levels of these molecules in the HJBE1-fed fish suggest that bioactive constituents within HJBE, particularly polyphenols, flavonoids, and vitamin C, stimulated immune cell activity or enhanced the synthesis of immune proteins. Previous research on phytogenic additives has reported similar effects, where plant extracts rich in antioxidants improved lysozyme and immunoglobulin responses in fish [7,11,12]. The mechanism underlying this immunostimulatory effect is likely multifactorial. First, the antioxidant potential of HJBE may have reduced oxidative stress within immune tissues. Excessive ROS can impair leukocyte function and antibody synthesis, while dietary antioxidants help preserve immune competence by stabilizing cellular membranes and protecting biomolecules from oxidation [38,39]. In the present study, the groups showing balanced antioxidant enzyme profiles (HJBE0.5 and HJBE1) also demonstrated higher immune indices, supporting the concept of an antioxidant–immune interaction. Second, polyphenolic compounds may act as mild immunomodulators, enhancing macrophage and lymphocyte activity through the activation of cytokine signaling pathways such as NF-κB and MAPK, which are known to regulate lysozyme and IgM gene expression [40,41]. The improved survival against *E. tarda* infection observed in the HJBE1 group further validates the physiological relevance of these immune enhancements. *E. tarda* is a well-known opportunistic pathogen in marine aquaculture, and resistance to infection is closely associated with the activation of immune defenses [42,43]. The significantly higher post-infection survival rate in fish fed HJBE1 indicates that the extract not only enhanced basal immune status but also improved the ability to mount an effective immune response under pathogenic challenge. Such protective effects of phytogenic extracts have been widely reported, where supplementation with fruit peel or herbal polyphenols enhanced survival against *Aeromonas hydrophila* and *Vibrio* spp. in multiple finfish species [44,45]. It is noteworthy that increasing the inclusion level beyond 1.0 g/kg did not further improve immune or survival outcomes. This plateau or decline in immunological response at higher dosages may be attributed to metabolic saturation or to potential inhibitory effects of excessive polyphenols, which can interfere with nutrient absorption or induce mild oxidative stress [46]. Thus, the immunostimulatory effects of HJBE appear to follow a dose-dependent, non-linear relationship similar to the trends observed in growth and antioxidant parameters.

## 5. Conclusions

In conclusion, dietary inclusion of 1.0 g/kg HJBE effectively enhanced the growth performance, digestive enzyme activity, antioxidant capacity, and immune response and improved disease resistance against *E. tarda* in juvenile *P. major*. However, higher inclusion levels did not provide additional benefits and may have even impaired physiological balance. These findings suggest that HJBE can serve as a promising natural and sustainable functional feed additive for improving fish health and performance while supporting the valorization of fruit-processing by-products in aquaculture.

## Figures and Tables

**Table 1:** Vitamin C, total phenolics, and total flavonoids contents, and radical scavenging activities of *Hylocereus polyrhizus* juice by-product extract (HJBE).

HJBE Composition								
Chemical compounds	Vitamin C (mg 100 g^−1^)	38.08 ± 1.245						
	Total phenolics (gallic acid mg 100 g^−1^)	17.20 ± 2.455						
	Total flavonoids (quercetin mg g^−1^)	14.66 ± 1.961						
Radical scavenging activities	Concentration (µg mL^−1^)	4000	2000	1000	500	250	125	IC_50_
	DPPH (%)	24.05	11.60	6.45	4.80	3.58	2.36	4.8
	ABTS (%)	22.77	10.63	4.82	3.42	2.11	1.96	5.0

Abbreviations: DPPH, 1,1–diphenyl–2–picrylhydrazyl; ABTS, 2,2′–azinobis–(3–ethylbenzothiazoline–6–sulfonate).

**Table 2:** Composition and proximate analysis of the experimental diets with different levels of *Hylocereus polyrhizus* juice by-product extract (HJBE) (expressed as g kg^−1^ in dry matter).

Parameters	Experimental Diets				
	HJBE0	HJBE0.5	HJBE1	HJBE2	HJBE3
Sardine meal	600	600	600	600	600
Dehulled soybean meal	120	120	120	120	120
Wheat flour	185	185	185	185	185
HJBE	0	0.5	1	2	3
Fish oil	35	35	35	35	35
Soybean oil	35	35	35	35	35
Vitamin premix ^a^	10	10	10	10	10
Mineral premix ^b^	10	10	10	10	10
Choline	5	5	5	5	5
Proximate composition (g kg^−1^, dry matter basis)					
Dry matter	97.0	97.3	97.5	97.8	97.7
Crude protein	51.0	50.7	50.8	50.9	51.0
Crude lipid	11.6	11.9	11.8	11.5	11.6
Ash	9.7	9.7	9.8	9.6	9.8

^a^ Vitamin premix contained the following amount, which was diluted in cellulose (g kg^−1^ mix): L-ascorbic acid, 121.2; DL-α-tocopheryl acetate, 18.8; thiamin hydrochloride, 2.7; riboflavin, 9.1; pyridoxine hydrochloride, 1.8; niacin, 36.4; Ca-D-pantothenate, 12.7; myo-inositol, 181.8; D-biotin, 0.27; folic acid, 0.68; p-aminobenzoic acid, 18.2; menadione, 1.8; retinyl acetate, 0.73; cholecalciferol, 0.003; cyanocobalamin, 0.003. ^b^ Mineral premix contained the following ingredients (g kg^−1^ mix): MgSO_4_·7H_2_O, 80.0; NaH_2_PO_4_·2H_2_O, 370.0; KCl, 130.0; ferric citrate, 40.0; ZnSO_4_·7H_2_O, 20.0; Ca-lactate, 356.5; CuCl, 0.2; AlCl_3_·6H_2_O, 0.15; KI, 0.15; Na_2_Se_2_O_3_, 0.01; MnSO_4_·H_2_O, 2.0; CoCl_2_·6H_2_O, 1.0.

**Table 3:** Growth performance and feed utilization of juvenile red seabream fed the experimental diets with different levels of *Hylocereus polyrhizus* juice by-product extract (HJBE) for 8 weeks.

Parameters	Experimental Diets					*p* Value
	HJBE0	HJBE0.5	HJBE1	HJBE2	HJBE3	
Initial weight (g fish^−1^)	7.0 ± 0.00	7.0 ± 0.00	7.0 ± 0.01	7.0 ± 0.00	7.0 ± 0.01	-
Final weight (g fish^−1^)	37.6 ± 0.33 ^a^	38.3 ± 0.85 ^a^	42.2 ± 0.30 ^b^	38.3 ± 1.04 ^a^	36.6 ± 1.62 ^a^	0.001
SR (%)	95.2 ± 1.90	93.3 ± 2.52	94.3 ± 1.65	91.4 ± 0.00	91.4 ± 0.00	0.387
WG (g fish^−1^)	30.7 ± 0.24 ^a^	31.3 ± 0.85 ^a^	35.2 ± 0.30 ^b^	31.3 ± 1.04 ^a^	28.6 ± 1.61 ^a^	0.001
SGR (%)	3.59 ± 0.019 ^a^	3.63 ± 0.048 ^a^	3.83 ± 0.016 ^b^	3.62 ± 0.058 ^a^	3.53 ± 0.092 ^a^	0.001
FI	35.1 ± 0.49 ^a^	37.4 ± 0.32 ^b^	40.0 ± 0.35 ^b^	37.0 ± 0.50 ^ab^	36.8 ± 0.0.42 ^ab^	<0.001
FE	0.92 ± 0.008 ^b^	0.90 ± 0.008 ^b^	0.93 ± 0.010 ^b^	0.93 ± 0.019 ^b^	0.85 ± 0.011 ^a^	0.002
PER	1.72 ± 0.038 ^b^	1.65 ± 0.058 ^ab^	1.73 ± 0.015 ^b^	1.66 ± 0.034 ^ab^	1.53 ± 0.020 ^a^	0.017
CF	1.95 ± 0.064	1.89 ± 0.050	1.94 ± 0.019	1.93 ± 0.007	1.92 ± 0.058	0.880
VSI (%)	1.73 ± 0.087	1.76 ± 0.056	1.80 ± 0.045	1.80 ± 0.157	1.81 ± 0.131	0.984
HSI (%)	1.96 ± 0.060	1.87 ± 0.024	1.91 ± 0.083	1.82 ± 0.020	1.75 ± 0.057	0.144

Values are means ± SE (n = 3), and values with different superscript letters within a row differ significantly (*p* < 0.05), while those without superscripts are not significantly different. Abbreviations: SR, survival; WG, weight gain; SGR, specific growth rate; FI, feed intake; FE, feed efficiency; PER, protein efficiency ratio; CF, condition factor; VSI, vicerosomatic index; HSI, hepatosomatic index.

**Table 4:** Digestive enzyme activities (mU mg^−1^ protein) of juvenile red seabream fed the experimental diets with different levels of *Hylocereus polyrhizus* juice by-product extract (HJBE) for 8 weeks.

Parameters	Experimental Diets					*p* Value
	HJBE0	HJBE0.5	HJBE1	HJBE2	HJBE3	
Amylase	37.9 ± 3.49	38.6 ± 3.30	37.4 ± 3.36	37.3 ± 3.99	37.6 ± 1.76	0.899
Trypsin	13.4 ± 1.62 ^a^	18.8 ± 1.00 ^ab^	22.4 ± 2.12 ^b^	17.2 ± 1.32 ^ab^	12.1 ± 0.93 ^a^	0.003
Lipase	134.8 ± 10.65	133.0 ± 16.07	160.1 ± 14.13	138.7 ± 12.68	132.6 ± 4.36	0.503

Values are means ± SE (n = 3), and values with different superscript letters within a row differ significantly (*p* < 0.05), while those without superscripts are not significantly different.

**Table 5:** Proximate composition (%) and hematological parameters of juvenile red seabream fed the experimental diets with different levels of *Hylocereus polyrhizus* juice by-product extract (HJBE) for 8 weeks.

Parameters	Experimental Diets					*p* Value
	HJBE0	HJBE0.5	HJBE1	HJBE2	HJBE3	
Moisture	67.5 ± 0.15	67.3 ± 0.15	67.6 ± 0.07	67.7 ± 0.15	67.5 ± 0.09	0.261
Crude protein	17.5 ± 0.15	17.8 ± 0.09	17.7 ± 0.21	17.7 ± 0.18	17.8 ± 0.17	0.745
Crude lipid	8.7 ± 0.32	8.5 ± 0.15	8.5 ± 0.20	8.7 ± 0.19	8.7 ± 0.09	0.936
Ash	4.6 ± 0.03	4.4 ± 0.12	4.4 ± 0.19	4.4 ± 0.09	4.3 ± 0.10	0.578
AST (U/L)	51.3 ± 9.24	55.0 ± 6.93	57.3 ± 8.11	51.7 ± 3.48	52.7 ± 2.73	0.960
ALT (U/L)	4.7 ± 0.33	5.0 ± 0.58	4.7 ± 0.33	5.3 ± 0.88	4.7 ± 0.33	0.866
TCHO (mg/dL)	355.0 ± 47.89	355.3 ± 29.36	361.0 ± 29.84	352.0 ± 32.62	354.7 ± 33.69	0.988
GLU (mg/dL)	87.3 ± 12.72	87. 3 ± 10.33	88.0 ± 9.61	88.0 ± 3.21	80.7 ± 4.33	0.969
TP (g/dL)	6.4 ± 1.23	6.0 ± 1.15	6.3 ± 1.11	6.3 ± 0.88	6.3 ± 0.88	0.984

Values are means ± SE (n = 3), and values without superscripts are not significantly different (*p* > 0.05). Abbreviations: AST, aspartate aminotransferase; ALT, alanine aminotransferase; TCHO, total cholesterol; GLU, glucose; TP, total protein.

**Table 6:** Plasma antioxidant enzyme activity of juvenile red seabream fed the experimental diets with different levels of *Hylocereus polyrhizus* juice by-product extract (HJBE) for 8 weeks.

Parameters	Experimental Diets					*p* Value
	HJBE0	HJBE0.5	HJBE1	HJBE2	HJBE3	
SOD (U/mL)	0.54 ± 0.062 ^a^	0.53 ± 0.036 ^a^	0.48 ± 0.022 ^a^	0.50 ± 0.044 ^a^	0.54 ± 0.044 ^a^	0.825
CAT (nmol/min/mL)	386.8 ± 39.51 ^c^	221.5 ± 11.97 ^a^	209.7 ± 0.78 ^a^	275.3 ± 8.71 ^ab^	366.1 ± 0.88 ^bc^	0.001
GSH (µM)	5.29 ± 0.109 ^b^	4.43 ± 0.182 ^a^	4.41 ± 0.215 ^a^	4.80 ± 0.177 ^ab^	5.35 ± 0.127 ^b^	0.005

Values are means ± SE (n = 3), and values with different superscript letters within a row differ significantly (*p* < 0.05), while those without superscripts are not significantly different. Abbreviations: SOD, superoxide dismutase; CAT, catalase; GSH, glutathione.

**Table 7:** Immune parameters of juvenile red seabream fed the experimental diets with different levels of *Hylocereus polyrhizus* juice by-product extract (HJBE) for 8 weeks.

Parameters	Experimental Diets					*p* Value
	HJBE0	HJBE0.5	HJBE1	HJBE2	HJBE3	
Lysozyme activity (U/mL)	0.080 ± 0.0043 ^a^	0.133 ± 0.0088 ^bc^	0.171 ± 0.0172 ^c^	0.122 ± 0.0107 ^ab^	0.109 ± 0.0051 ^ab^	0.001
IgM (mg/mL)	225.3 ± 10.92 ^a^	231.8 ± 12.99 ^a^	292.0 ± 7.58 ^b^	264.5 ± 5.99 ^ab^	237.5 ± 10.33 ^a^	0.004

Values are means ± SE (n = 3), and values with different superscript letters within a row differ significantly (*p* < 0.05), while those without superscripts are not significantly different. Abbreviations: IgM, immunoglobulin M.

## Data Availability

The original contributions presented in this study are included in the article. Further inquiries can be directed to the corresponding author.
